# Supplementary material for: Healthcare Utilization and Costs after Receiving a Positive BRCA1/2 Result from a Genomic Screening Program
Source: J Pers Med. 2020 Feb 3;10(1):7. doi: 10.3390/jpm10010007 (PMC7151600; doi:10.3390/jpm10010007)
Supplement: Supplementary file 1 [file jpm-10-00007-s001.pdf]

Table S1. Codes Used to Identify *BRCA1/BRCA2* Risk-Reducing Surgical Procedures, Imaging Procedures, and Chemoprevention

Procedure	Codes	Code System
**Mastectomy**	13101, 19126, 19296, 19297, 19300, 19301, 19302, 19303, 19304, 19305, 19306, 19307, 19316	CPT
**Oophorectomy**	49320, 58150, 58200, 58262, 58542, 58544, 58550, 58570, 58571, 58573, 58660, 58661, 58662, 58720, 58940, 58943, 58950, 58953, 58956, 59150, 59151	CPT
**Magnetic Resonance Imaging (MRI)**	76093, 77058, 19085, 76094, 77058, 77059, 19287, 76094, 77021	CPT
**Mammography**	76090, 76091, 76092, 77051, 77052, 77055, 77056, 77057, 77061, 77062, 77063, 77065, 77066, 77067, G0202, G0204, G0206, G0279	CPT
**Chemoprevention**	V07.51	ICD-9
	Z79.810	ICD-10
	202735, 202677, 541010, 229467, 41851, 15095, 540356, 31102, 540992, 227723, 20941, 229249, 227794, 202570, 202519, 229250, 42652, 42646, 201712, 229251, 208555, 229252, 208748	Medication Identification Number

Note: Additional system-specific Electronic Health Record (EHR) codes were applied in some cases.
CPT, Current Procedural Terminology.
